# Clinical outcomes and inflammatory profiles in culture-positive sepsis: A retrospective cohort study

**DOI:** 10.1177/03000605261454273

**Published:** 2026-08-25

**Authors:** Nuril Farid Abshori, Khoirunnisa Luthfiyyah Andarwati, Fatahillah Tsabit Fatoni, Tio Rizki Fauzi, Achmad Zainudin Arif, Iwal Reza Ahdi, Muhammad Habiburrahman

**Affiliations:** 1 Faculty of Medicine and Health Sciences, 232278 Maulana Malik Ibrahim Islamic State University Malang, Malang, Indonesia; 2 Medical Student Program Faculty of Medicine and Health Sciences, 232278 Maulana Malik Ibrahim Islamic State University, Malang, Indonesia; 3 Bachelor of Pharmacy Program Faculty of Medicine and Health Sciences, 232278 Maulana Malik Ibrahim Islamic State University, Malang, Indonesia; 4 Department of Internal Medicine, Faculty of Medicine and Health Sciences, 232278 Maulana Malik Ibrahim Islamic State University Malang, Malang, Indonesia; 5 Faculty of Medicine, 4615 Universitas Indonesia, Jakarta, Indonesia; 6 Faculty of Medicine, Imperial College London, London, UK

**Keywords:** Sepsis, gram-negative bacteria, gram-positive bacteria, neutrophil-to-lymphocyte ratio, platelet-to-lymphocyte ratio, mortality, inflammatory biomarkers

## Abstract

**Objective:**

To describe clinical outcomes and inflammatory profiles in patients with culture-positive sepsis identified using Sepsis-3 criteria and to explore their association with bacterial Gram stain status.

**Methods:**

A retrospective cohort study was conducted in adult patients with sepsis hospitalized at a single-center hospital in East Java, Indonesia, in 2023. Patients were identified using Sepsis-3 criteria and included if blood cultures were obtained at admission. Data collected included blood culture results, neutrophil-to-lymphocyte ratio, platelet-to-lymphocyte ratio, comorbidities, length of hospital stay, and in-hospital mortality. Patients were grouped according to bacterial Gram stain status. Group differences were analyzed using the chi-square test for categorical variables and the Mann–Whitney U test for continuous variables. Multivariate logistic regression was performed to identify factors associated with mortality.

**Results:**

Among 172 patients clinically diagnosed with sepsis, blood culture was positive in 46 cases (26.7%), indicating that most patients (73.3%) had culture-negative sepsis. Among culture-positive cases, gram-positive organisms predominated (n = 37), whereas gram-negative pathogens were less frequently isolated (n = 9). Increased inflammatory burden, reflected by a higher admission neutrophil-to-lymphocyte ratio, was associated with worse clinical outcomes. In multivariate analysis, gram-negative infection (adjusted odds ratio = 4.00, 95% confidence interval: 1.05–15.30), higher neutrophil-to-lymphocyte ratio (adjusted odds ratio = 1.06 per unit; 95% confidence interval: 1.01–1.11), and acute kidney injury (adjusted odds ratio = 5.30, 95% confidence interval: 1.73–16.50) were independently associated with in-hospital mortality.

**Conclusion:**

Culture-negative sepsis was common, likely influenced by early antibiotic use. Gram-positive pathogens predominated among culture-positive cases, whereas gram-negative infections were associated with worse clinical outcomes. Elevated neutrophil-to-lymphocyte ratio and acute kidney injury independently predicted in-hospital mortality. These findings support the need for multimodal sepsis assessment integrating clinical judgment and laboratory markers rather than relying solely on culture results.

## Introduction

Sepsis is a life-threatening organ dysfunction resulting from a dysregulated host inflammatory response to infection.^1^ The global burden of sepsis remains high. The incidence of hospital-treated sepsis has been estimated at 189 cases per 100,000 person-years, with a mortality rate of 16.7%, while the incidence of sepsis requiring intensive care unit (ICU) admission was 58 cases per 100,000 person-years, of which 41.9% of patients died before hospital discharge.^2^ Similar patterns have been observed in Indonesia, where approximately one-third of ICU admissions are sepsis-related.^3^ Both gram-negative bacteria, such as *Escherichia coli*, *Klebsiella pneumoniae*, and *Pseudomonas aeruginosa*, and gram-positive bacteria, such as *Staphylococcus aureus* and *Streptococcus pneumoniae*, are leading etiologic agents of sepsis.^4^

In recent decades, gram-positive bacteria have become the predominant cause of sepsis, a trend attributed to increased use of invasive medical devices, prolonged hospitalization, and the emergence of antibiotic resistance.^5^ Despite their higher prevalence, clinical outcomes in patients with gram-positive sepsis are often heterogeneous, prompting continued investigation into factors that differentiate disease severity across bacterial groups. In contrast, gram-negative pathogens, although less frequently isolated, are more commonly associated with severe shock and heightened systemic inflammatory responses.^6^ Although several meta-analyses have reported no significant difference in 28-day mortality between gram-positive and gram-negative sepsis,^6^ gram-negative infections are consistently associated with more severe inflammation, characterized by elevated interleukin-6 (IL-6) and IL-10 levels.^7^ This exaggerated response is primarily driven by lipopolysaccharide (LPS), a gram-negative endotoxin that potently activates innate immune pathways, contributing to multiorgan dysfunction and increased mortality risk.^8^

Beyond microbiological classification, simple hematological biomarkers play an essential role in prognostic stratification of sepsis.^9^ The neutrophil-to-lymphocyte ratio (NLR) reflects the balance between innate immune activation and adaptive immune suppression and has been widely associated with disease severity and mortality.^10^ A large meta-analysis involving more than 10,000 patients with sepsis demonstrated that elevated NLR was significantly associated with increased mortality (hazard ratio = 1.68).^11^ In contrast, the prognostic value of the platelet-to-lymphocyte ratio (PLR) remains inconclusive: some studies have reported an association with mortality,^12^ while others have found no significant prognostic value.^10^

Accordingly, this study aimed to evaluate the clinical impacts of gram-positive versus gram-negative bacterial infections on mortality, length of hospital stay (LOS), and inflammatory indices (NLR and PLR) among patients with sepsis identified using Sepsis-3 criteria at Karsa Husada Hospital, Batu, Indonesia.

## Methods

### Study design and population

**Figure 1.**
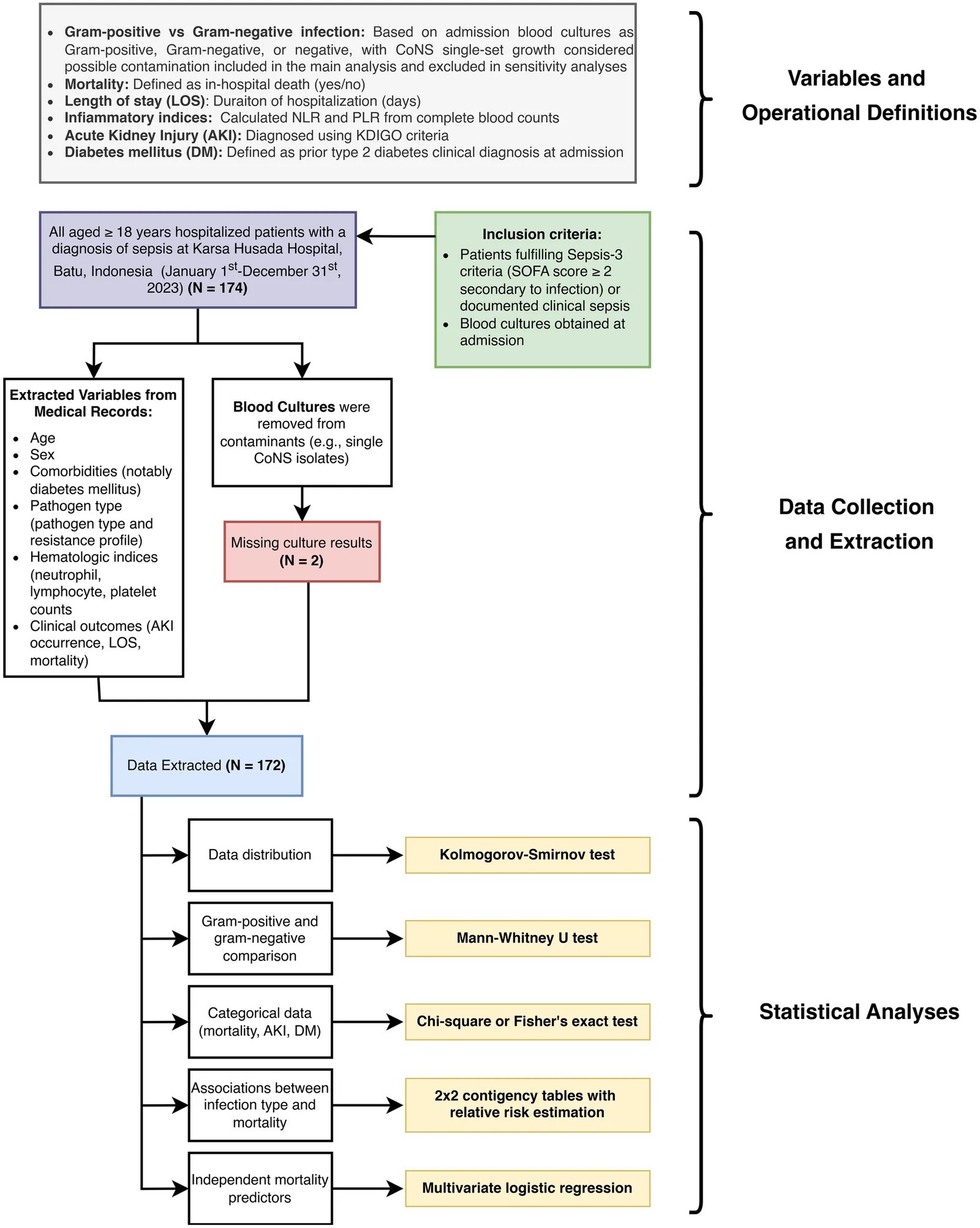
Study flowchart and analytical framework. Overview of the methodological workflow of this retrospective cohort study at Karsa Husada Hospital, Batu, Indonesia (January–December 2023). Adult patients (≥18 years) diagnosed with sepsis based on Sepsis-3 criteria (SOFA score ≥2 or documented clinical sepsis) were included. Blood cultures obtained at admission were used to classify infections as gram-positive or gram-negative; contaminants (e.g., single-set coagulase-negative *Staphylococci*) and missing culture data (n = 2) were excluded, leaving 172 patients with sepsis for analysis. Extracted variables included demographics, comorbidities (notably diabetes mellitus), pathogen type, hematologic indices (for neutrophil-to-lymphocyte ratio and platelet-to-lymphocyte ratio calculation), and clinical outcomes (AKI, length of stay, and mortality). Statistical analyses comprised the Kolmogorov–Smirnov test for data distribution, Mann–Whitney U test for continuous variables, chi-square or Fisher's exact test for categorical data, 2 × 2 contingency tables for relative risk estimation, and multivariate logistic regression to identify independent mortality predictors.

This study employed a retrospective cohort design (Figure 1). The source population comprised all consecutively admitted adult patients (aged ≥18 years) hospitalized with sepsis at Karsa Husada Hospital, Batu, Indonesia, between 1 January and 31 December 2023. Inclusion criteria were as follows: (a) patients fulfilling Sepsis-3 criteria (Sequential Organ Failure Assessment (SOFA) score ≥2 secondary to infection) or documented clinical sepsis and (b) blood cultures obtained at admission. Patients with incomplete culture or laboratory data were excluded. Of the 174 sepsis cases identified, 2 were excluded because of missing culture results, yielding 172 patients with complete data for analysis. The conduct and reporting of this retrospective cohort study were performed in accordance with the Strengthening the Reporting of Observational Studies in Epidemiology (STROBE) guidelines.^13^

AKI: acute kidney injury.

### Variables and outcome measurements

Classification of bacteremia was based on the results of blood cultures obtained in two sets from separate venous draws at hospital admission. A culture was considered positive if pathogenic bacteria grew, and the organism was further categorized as gram-positive or gram-negative based on its characteristics. In cases with more than one isolate, a single category was assigned by selecting the isolate considered the dominant clinical pathogen to avoid multiple counts from a single case. The growth of coagulase-negative *Staphylococcus* (CoNS) in one set of cultures was considered possible contamination; this finding was considered gram-positive in the main analysis and excluded in the sensitivity analysis. Cultures without bacterial growth were classified as negative. Because the gram-negative subgroup was small (n = 9), imprecise estimates, wide confidence intervals (CIs), and an increased risk of overestimation may have resulted. Therefore, simple models and sensitivity analyses were considered to assess the robustness of the findings.

For statistical analysis, the main outcomes included in-hospital mortality during the acute care period, measured as a yes/no outcome, and LOS, calculated in days from admission to discharge or death. Inflammatory indices, namely NLR and PLR, were calculated from the complete blood count obtained on the first day of treatment. Acute kidney injury (AKI) was diagnosed based on medical records following Kidney Disease: Improving Global Outcomes (KDIGO) criteria: an increase in serum creatinine of ≥0.3 mg/dL within 48 h or ≥1.5× baseline levels during treatment. Diabetes mellitus (DM) was defined as a pre-existing clinical diagnosis of type 2 DM documented upon admission.

### Data collection

Data were retrieved from the hospital information system and patient medical records under institutional permission. Extracted variables included demographics (age and sex), comorbidities (notably DM), microbiology results (pathogen type and resistance profile), hematologic indices (neutrophil, lymphocyte, and platelet counts), and clinical outcomes (AKI occurrence, LOS, and mortality). Blood cultures were processed in the hospital microbiology laboratory, and the investigators reviewed potential contaminants (e.g. single CoNS isolates). All data were anonymized and securely stored.

### Ethical considerations

This study was conducted in accordance with the ethical principles of the Declaration of Helsinki (1975), as revised in 2024. Ethical approval was obtained from the Health Research Ethics Committee of the Faculty of Medicine and Health Sciences, Maulana Malik Ibrahim Islamic State University Malang, in collaboration with Karsa Husada General Hospital, Batu, Indonesia (Approval No. 100/03/EC/KEPK-FKIK/06/2025; approval date: 3 June 2025). Given the retrospective, noninterventional study design and the use of anonymized medical records, the ethics committee waived the requirement for written informed consent (Letter No. 009/KEPK.FKIK/KP/XI/2025). All patient data were fully de-identified prior to analysis, and no information permitting individual patient identification was accessible to the investigators. Artificial intelligence–assisted tools were used solely for language editing and clarity improvement. All scientific content, data analysis, interpretation, and final editorial decisions were performed by the authors.

### Statistical analysis

All analyses were performed using SPSS version 26. Data distribution was assessed using the Kolmogorov–Smirnov test. Continuous variables were summarized as median and interquartile range (IQR) because of skewed distributions and were compared using nonparametric tests. Specifically, NLR, PLR, and LOS were compared between gram-positive and gram-negative groups using the Mann–Whitney U test. Categorical data (mortality, AKI, and DM) were expressed as frequencies (%) and analyzed using chi-square tests. Because the continuous variables were not normally distributed, the Student's *t*-test was not used. Instead, the Mann–Whitney U test was applied for comparisons between independent groups.

Associations between infection type (gram-negative vs. gram-positive) and mortality were evaluated using 2 × 2 contingency tables with relative risk (RR) estimation. To identify independent predictors of mortality, multivariate logistic regression was performed, including infection type, NLR, PLR, AKI, and DM as covariates. Age and sex were excluded to maintain model parsimony, given their balanced distribution and lesser clinical influence in this dataset. The enter method was applied. Results were expressed as adjusted odds ratios (aORs) with 95% CIs. Statistical significance was set at p < 0.05 (two-tailed).

### Sensitivity analysis

To account for potential CoNS contamination, the main analysis was repeated after excluding patients with single-set CoNS cultures. This analysis assessed whether excluding likely contaminants altered the primary associations. All analyses were two-sided.

## Results

### Patient characteristics

In 2023, 172 patients with sepsis met the study inclusion criteria. The mean age was 61 ± 16 years (range, 19–89 years), and 54% were female. A total of 58 patients (33.7%) had type 2 DM as a comorbidity. The incidence of AKI during hospitalization was relatively high, occurring in 45 patients (26.2%). The overall in-hospital mortality rate was 34.3% (59 deaths). Table 1 summarizes the key demographic and clinical characteristics of the study population, including the distribution of infection types based on blood culture results.

**Table 1.** Demographic and clinical characteristics of patients with sepsis and distribution of blood culture results. “No growth” indicates negative cultures. Gram-positive versus gram-negative classification was based on the predominant pathogenic organism.

Characteristic	Overall (N = 172)	Gram-positive (n = 37)	Gram-negative (n = 9)	Negative culture (n = 126)
Age, years (median, IQR)	62 (50–72)	64 (52–75)	59 (48–68)	61 (49–72)
Sex, M/F (%)	54%/46%	51%/49%	56%/44%	55%/45%
Diabetes mellitus (%)	33.7%	35.1%	33.3%	33.3%
AKI during treatment (%)	26.2%	21.6%	44.4%	25.4%
Length of hospital stay, days (median)	8 (5–15)	7 (4–14)	10 (6–18)	8 (5–15)
In-hospital mortality (%)	34.3%	29.7%	55.6%	33.3%
Blood test results:				
Gram-positive (%)	21.5% (37 patients)	100%	0%	0%
Gram-negative (%)	5.2% (9 patients)	0%	100%	0%
No growth (negative) (%)	73.3% (126 patients)	0%	0%	100%
Common bacterial species:				
*Staphylococcus* coagulase-negative	–	22 (59.5%)	–	–
*Streptococcus spp.*	–	5 (13.5%)	–	–
*Staphylococcus aureus*	–	3 (8.1%)	–	–
*Enterococcus faecalis*	–	2 (5.4%)	–	–
*Bacillus spp.* (contaminant)	–	2 (5.4%)	–	–
*Escherichia coli*	–	–	4 (44.4%)	–
*Klebsiella spp.*	–	–	3 (33.3%)	–
*Pseudomonas aeruginosa*	–	–	1 (11.1%)	–
Others	–	–	1 (11.1%)	–

CoNS: coagulase-negative *Staphylococci* (e.g. *S. epidermidis* and *S. haemolyticus*), commonly considered skin contaminants. IQR: interquartile range.

**Figure 2.**
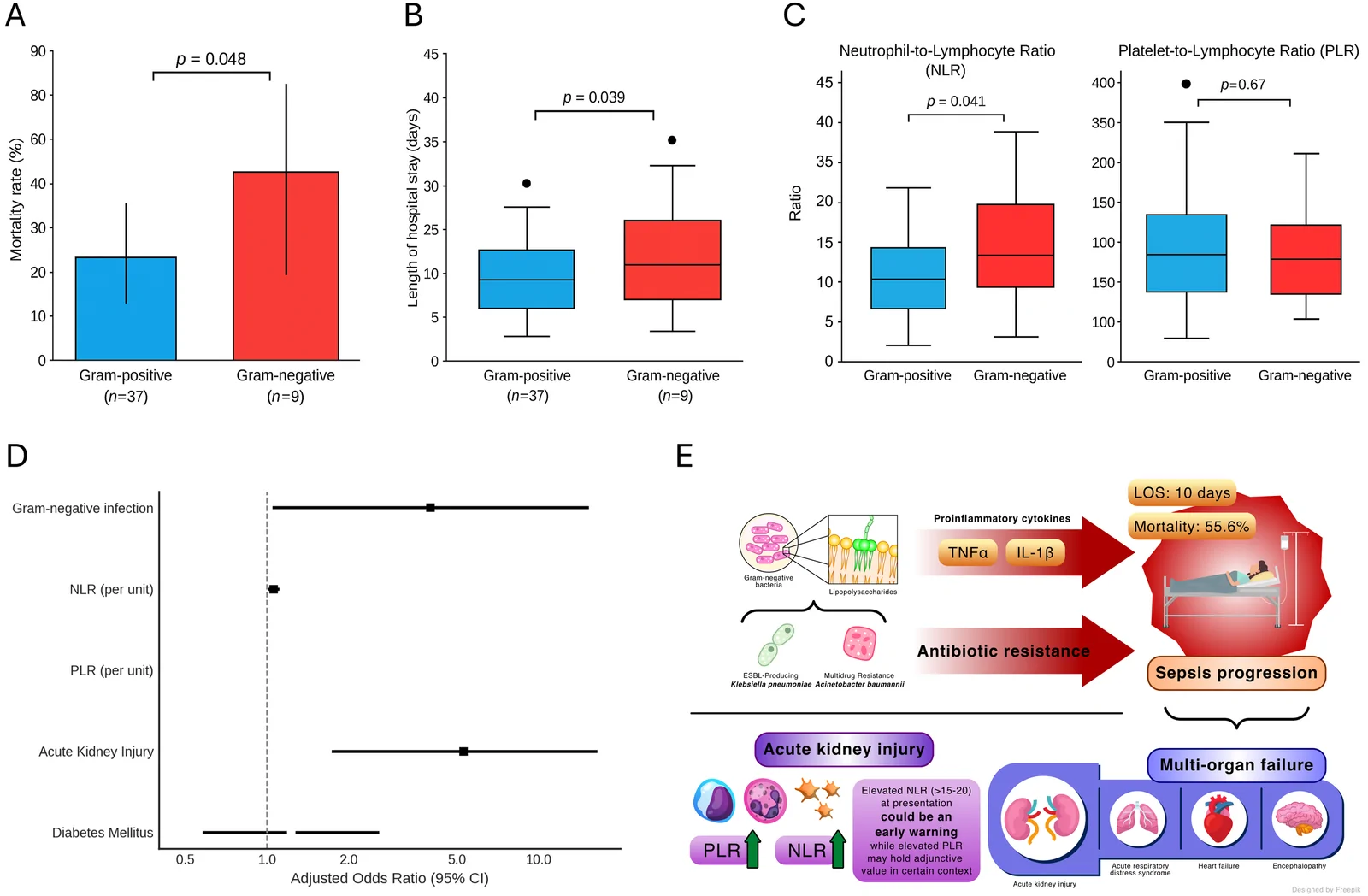
(a) Gram-negative sepsis showed higher mortality (55.6%, 5/9) compared with gram-positive sepsis (29.7%, 11/37; p = 0.048, χ^2^ test), with an approximately twofold increased risk of death. (b) Hospital stay was longer in patients with gram-negative infections (median 10 days vs. 7 days; p = 0.039, Mann–Whitney U test), indicating a more severe disease. (c) The neutrophil-to-lymphocyte ratio (NLR) was higher in gram-negative infections (16.8 vs. 10.4; p = 0.041), while the platelet-to-lymphocyte ratio (PLR) was similar between the two groups (p = 0.67). (d) Multivariate analysis showed that gram-negative infection (aOR = 4.0), high NLR (aOR = 1.06/unit), and AKI (aOR = 5.3) were associated with increased mortality, whereas PLR and diabetes mellitus were not significant. (e) Gram-negative bacteria, particularly *Klebsiella pneumoniae* and *Acinetobacter baumannii*, contain lipopolysaccharides that trigger the release of TNF-α and IL-1β, leading to excessive inflammatory responses, antibiotic resistance, longer hospital stays, and higher mortality. Increased NLR and PLR reflect systemic inflammation and serve as prognostic biomarkers in gram-negative sepsis.

Among 172 patients with suspected sepsis, only 46 (26.7%) had culture-confirmed infections, underscoring the predominance of culture-negative sepsis in this cohort. As illustrated in Figure 2(a), patients with gram-negative sepsis demonstrated significantly higher in-hospital mortality than those with gram-positive sepsis (p = 0.048). Furthermore, as shown in Figure 2(b), the median hospital stay was significantly longer in the gram-negative group (10 days) than in the gram-positive group (7 days; p = 0.039), suggesting more severe illness or complications in patients with gram-negative infections. Gram-positive infections (37 patients) were most frequently caused by CoNS (22 cases). Although CoNS can act as opportunistic pathogens in immunocompromised hosts, they are more often regarded as contaminants. Other gram-positive isolates included *Streptococcus* spp. (5 cases, potentially including *S. pneumoniae*), *Staphylococcus aureus* (3 cases), and *Enterococcus faecalis* (2 cases). Two gram-positive cultures yielded *Bacillus* spp., likely representing environmental contaminants. In contrast, gram-negative infections (9 patients) were primarily due to *Escherichia coli* (4 cases), followed by *Klebsiella* spp. (3 cases), and single isolates of *Pseudomonas aeruginosa* and *Edwardsiella ictaluri*.

AKI: acute kidney injury; IL-1β: interleukin-1β; TNF-α: tumor necrosis factor-α.

### Inflammatory indices (NLR and PLR) and LOS

Baseline NLR values varied substantially among patients (median 10.8; IQR: 5.5–18.9). As shown in Figure 2(c), when stratified by bacterial group, patients with gram-negative infections had significantly higher NLR levels than those with gram-positive infections (median 16.8 (IQR: 9.2–26.1) vs. 10.4 (IQR: 5.1–15.6); p = 0.041), indicating a more intense inflammatory response in gram-negative sepsis. In contrast, the median PLR did not differ significantly between groups (approximately 150 vs. 140; p = 0.67). Moreover, the correlation between PLR and NLR was weak (Spearman ρ = 0.08; p = 0.21), suggesting that PLR is less sensitive in reflecting inflammatory variation across bacterial categories in this cohort.

As described in Table 2, LOS ranged from 1 to 45 days, with a median of 8 days. Patients with gram-negative infections had longer hospitalizations than those with gram-positive infections (median 10 vs. 7 days; p = 0.039). This extended LOS in the gram-negative group likely reflects greater disease severity and higher complication rates. Patients with negative blood cultures had a median LOS of 8 days, comparable to the overall cohort median.

**Table 2.** Comparison of inflammatory markers and clinical outcomes between gram-positive and gram-negative sepsis.

Parameters	Gram-positive (n = 37)	Gram-negative (n = 9)	p value
Neutrophil-to-lymphocyte ratio (NLR), median (IQR)	10.4 (5.1–15.6)	16.8 (9.2–26.1)	0.041^a^
Platelet-to-lymphocyte ratio (PLR), median (IQR)	140 (85–205)	150 (90–220)	0.67
Spearman correlation (NLR–PLR)	ρ = 0.08	–	0.21
Length of stay (days), median (IQR)	7 (5–10)	10 (7–18)	0.039^a^

Comparisons between groups were performed using the Mann–Whitney U test; Spearman's rank correlation was used to assess the relationship between NLR and PLR. Data are presented as median (interquartile range). ^a^p < 0.05 was considered statistically significant.

### Mortality and logistic regression analysis

As illustrated in Figure 2(a), among 37 patients with gram-positive infections, 11 (29.7%) died during hospitalization. In contrast, mortality reached 55.6% (5/9) among patients with gram-negative infections, representing a statistically significant difference (χ^2^ test, p = 0.048). The RR of death in gram-negative sepsis was approximately 1.87, indicating an approximately twofold increase compared with gram-positive infections. In the culture-negative group, mortality was 33.3% (42/126), comparable to that of the overall cohort.

Other variables associated with mortality in univariate analyses included AKI and NLR. Mortality was markedly higher among patients with AKI (57.8%) than among those without AKI (23.3%; p < 0.001; RR = 2.48). Similarly, NLR values were significantly higher in nonsurvivors than in survivors (median 15.2 vs. 8.7; p = 0.002, Mann–Whitney U test). In contrast, DM was not significantly associated with mortality (36.2% vs. 33.3%; p = 0.75), nor were age or sex.

A multivariate logistic regression model was constructed to identify independent predictors of mortality, incorporating infection type (gram-negative vs. gram-positive), NLR, PLR, AKI, and DM. The final model (Table 3) was statistically significant (p < 0.001) and demonstrated good calibration (Hosmer–Lemeshow p = 0.46), explaining 35% of the variance in mortality (Nagelkerke R^2^ = 0.35). Gram-negative infection (aOR = 4.00, 95% CI: 1.05–15.30), higher NLR (aOR = 1.06 per unit, 95% CI: 1.01–1.11), and AKI (aOR = 5.30, 95% CI: 1.73–16.50) emerged as independent predictors of in-hospital mortality.

**Table 3.** Multivariate logistic regression for sepsis mortality.

Variable	Coefficient (β)	SE (β)	Adjusted OR (95% CI)	p value	Category (yes/no/positive/negative)
Gram-negative infection (vs. positive)	+1.386	0.677	4.00 (1.05–15.30)	0.041^a^	Negative/positive
NLR (per unit increase)	+0.058	0.026	1.06 (1.01–1.11)	0.025^a^	Continuous (median 10.8)
PLR (per unit increase)	+0.001	0.004	1.00 (0.99–1.01)	0.789	Continuous (median 145)
AKI during treatment (yes vs. no)	+1.667	0.578	5.30 (1.73–16.50)	0.004^b^	Yes/No
Diabetes mellitus (yes vs. no)	+0.210	0.383	1.23 (0.58–2.59)	0.580	Yes/No

^a^p < 0.05; ^b^p < 0.01. CI: confidence interval; OR: odds ratio; SE: standard error. The model included 172 observations; Hosmer–Lemeshow test p = 0.46 (indicating good fit); Nagelkerke R^2^ = 0.35.

As illustrated in Figure 2(d), in multivariate analysis, gram-negative infection conferred an aOR of approximately 4.0 (95% CI ± 2.5; p = 0.041) relative to gram-positive infection, indicating that after adjustment for confounders, patients with gram-negative bacteremia had approximately fourfold higher odds of death. Although the wide CI reflects the limited number of gram-negative cases, the direction and magnitude of the effect remained consistent. NLR was also independently associated with mortality (p = 0.025; aOR = 1.06 per 1-unit increase, 95% CI: 1.01–1.11), suggesting that each incremental rise in NLR corresponded to a 6% higher odds of death; for instance, a patient with an NLR of 20 would have approximately twice the odds of in-hospital mortality with an NLR of 10. AKI emerged as the strongest independent predictor of death (p = 0.004; aOR = 5.3, 95% CI: 1.7–16.5), with affected patients exhibiting a fivefold increase in mortality risk compared with those without AKI, independent of infection type and inflammatory indices, consistent with established evidence that renal dysfunction exacerbates sepsis outcomes. PLR showed no prognostic significance (p = 0.79; aOR  = 1.00), indicating minimal additive information beyond NLR, and DM was likewise not an independent predictor of mortality (p = 0.58; aOR  = 1.2).

## Discussion

A high proportion of patients in this cohort presented with culture-negative sepsis, a phenomenon widely reported in contemporary sepsis literature and reflective of real-world clinical practice rather than diagnostic inaccuracy. Early initiation of empirical antibiotics, often lifesaving in patients with sepsis, frequently precedes blood culture acquisition and substantially reduces microbiological yield. In addition, blood culture sensitivity is inherently limited by sampling volume, timing, and prior antimicrobial exposure.^14,15^ Finally, sepsis may arise in the absence of overt bacteremia, including in localized infections, infections with fastidious organisms, or partially treated infections.^16^ Collectively, these factors explain why negative culture results do not preclude a real septic process.

To ensure clinical relevance, all included patients met Sepsis-3 criteria, supporting the interpretation that culture-negative cases reflected true systemic infection. To the best of our knowledge, limited data from Indonesia have examined the combined association between Gram stain status, inflammatory indices (NLR/PLR), and clinical outcomes in patients with sepsis. Among culture-positive patients, gram-positive organisms predominated, consistent with hospital-based epidemiology in regions with high device utilization and prolonged inpatient care.^17^ Although contamination, particularly with CoNS, cannot be entirely excluded, restricting inclusion to patients with clinical sepsis likely mitigated this risk. Nonetheless, the predominance of gram-positive isolates primarily reflects local microbiological patterns rather than methodological bias.^18^

Although less frequent, gram-negative sepsis was associated with a substantially more severe clinical course. Mortality in the gram-negative group was nearly double that observed in patients with gram-positive infections, accompanied by longer hospital stays.^19^ This disproportionate fatality is biologically plausible, as gram-negative pathogens release LPS endotoxin, particularly the lipid A moiety, which acts as a potent trigger of innate immune activation and cytokine storm. The resulting cascade of systemic inflammation and endothelial dysfunction predisposes patients to septic shock and early multiorgan failure, explaining why gram-negative infections, although less common, are more lethal.^20^

Beyond pathogen classification, host inflammatory response and organ dysfunction emerged as central determinants of outcome. The higher NLR observed in gram-negative infections is driven by a strong neutrophil-mediated inflammatory response induced by gram-negative pathogens, primarily through LPS-mediated activation of innate immune signaling pathways. LPS stimulates cytokine release (including tumor necrosis factor-α (TNF-α), IL-6, and IL-1β), promotes neutrophil recruitment, and accelerates lymphocyte apoptosis, leading to both neutrophilia and relative lymphopenia, thereby increasing NLR.^21,22^ In contrast, PLR did not demonstrate prognostic value, suggesting that platelet-related inflammatory pathways may play a less prominent role in short-term mortality than neutrophil–lymphocyte imbalance.^23^ AKI was the strongest independent predictor of death, reinforcing its role as an early marker of systemic decompensation and irreversible disease progression.^4^

The predominance of CoNS can be explained by their strong association with healthcare-related procedures, particularly intravascular devices, frequent venipuncture, and prolonged hospital exposure. CoNS are also common skin commensals, which increases the likelihood of contamination during blood culture collection, especially when strict aseptic technique is difficult to maintain in routine clinical settings. In contrast, *Escherichia coli* and *Klebsiella pneumoniae* are typical gram-negative pathogens frequently linked to urinary tract infections, intra-abdominal infections, and hospital-acquired pneumonia, which are major sources of sepsis in hospitalized patients. Similar findings have been reported in other sepsis surveillance studies, where CoNS were among the most frequently isolated organisms in blood cultures, particularly in hospital-based cohorts with high device utilization and ICU exposure.^24^

From a clinical standpoint, the present study suggests the importance of careful clinical monitoring and individualized management in patients with gram-negative infections, which were associated with a higher frequency of adverse outcomes, including shock and in-hospital mortality, highlighting the need for timely empiric antimicrobial therapy with adequate gram-negative coverage, particularly in patients with suspected nosocomial or abdominal sources. Such an approach remains consistent with international recommendations, including the Surviving Sepsis Campaign (2021), which emphasizes early administration of appropriate broad-spectrum antibiotics.^25^ In contrast, narrower-spectrum therapy may be considered in community-acquired infections with a predominance of gram-positive pathogens, provided that microbiological data allow for de-escalation.^26^

This study has several limitations that should be acknowledged. First, the number of gram-negative cases was small (n = 9), limiting statistical precision and resulting in wide CIs. Therefore, the associations observed for gram-negative infection should be interpreted with caution and considered exploratory. Second, a substantial proportion of patients had culture-negative sepsis (73%), a finding that reflects real-world clinical practice in critical care settings and may be related to prior antibiotic exposure, limitations in microbiological diagnostics, and variability in the timing of culture acquisition. Empirical antibiotics may have been administered before blood culture collection in some patients, potentially reducing microbiological yield and contributing to the high proportion of culture-negative results. Consequently, the present findings apply primarily to culture-positive sepsis and may not fully represent outcomes in patients with culture-negative sepsis. Third, although analysis excluding potential CoNS contaminants yielded consistent results, misclassification cannot be entirely excluded, as blood culture results were interpreted alongside Sepsis-3 criteria rather than serving as the sole diagnostic determinant. Additionally, although admission NLR was associated with mortality, an optimal ROC-derived cutoff could not be determined due to the limited number of culture-positive cases. Finally, as a single-center retrospective study, generalizability is limited; however, the observed bacterial distribution, predominantly *Escherichia coli*, *Klebsiella* spp., and CoNS, is consistent with reports from other regional hospitals in Indonesia, supporting the relevance of these exploratory associations.

## Conclusion

A high proportion of patients in this cohort presented with culture-negative sepsis, reflecting real-world clinical practice rather than diagnostic inaccuracy. This finding is plausibly explained by early antibiotic administration. The inclusion of only Sepsis-3 criteria-defined patients with sepsis supports the interpretation that culture-negative cases represented true systemic infection. Among culture-positive cases, gram-positive organisms predominated, consistent with hospital-based epidemiology in settings characterized by prolonged hospitalization and high device utilization. Despite their lower frequency, gram-negative infections may be associated with a more severe clinical course, including nearly double the mortality and longer hospital stays compared with gram-positive sepsis. Compared with gram-positive sepsis, gram-negative sepsis was associated with longer in-hospital stays, while elevated admission NLR was associated with in-hospital mortality. AKI was strongly associated with mortality. These findings underscore the need for a cautious, multimodal approach to sepsis diagnosis, in which microbiological data are interpreted alongside clinical presentation and supportive laboratory markers, rather than being relied upon in isolation, especially in culture-negative sepsis.
